# Enhancing SNR in MRI at 7T using wearable coils, dielectric resonators, and dipole antennas

**DOI:** 10.1007/s10334-026-01323-0

**Published:** 2026-01-20

**Authors:** Daniel Wenz, Jules Vliem, Elizaveta Shegurova, Mark Widmaier, Lijing Xin, Dimitrios C. Karampinos, Irena Zivkovic

**Affiliations:** 1 CIBM Center for Biomedical Imaging, Lausanne, Switzerland; 2 MR Imaging and Technology, EPFL Swiss Federal Institute of Technology, Lausanne, Switzerland; 3 Department of Electrical Engineering, Eindhoven University of Technology, Eindhoven, The Netherlands; 4 Laboratory of Magnetic Resonance Imaging Systems and Methods, EPFL Swiss Federal Institute of Technology, Lausanne, Switzerland; 5 Institute of Physics (IPHYS), EPFL Swiss Federal Institute of Technology, Lausanne, Switzerland

**Keywords:** Flexible RF coil, Wearable RF coil, RF array, 7T, High-field MRI, UHF, Dipole antenna, Dielectric resonator, High-permittivity material

## Abstract

**Motivation:**

The twisted-pair (TP) coil design is a promising strategy for developing novel, flexible, wearable MRI detectors that can provide SNR gains in various clinical applications of high-field MRI. We hypothesize that the TP coil’s receive (Rx) sensitivity can be significantly increased by combining it with two complementary elements, such as dielectric resonators (DRs) and dipole antennas.

**Methods:**

TP coils were combined with DRs made of high-permittivity material (*ε*_r_ = 1070) and transceiver (TxRx) dipole antennas. The Tx and Rx performance of six different types of arrays (TP-only, dipole-only, TP with DRs, dipole with DRs, dipole with TPs, and dipole with TPs and DRs) was investigated through numerical simulations involving a cylindrical phantom suitable for lower extremity applications and two human voxel models. MR phantom experiments were conducted using a 7 Tesla whole-body MRI scanner to validate the Tx and Rx performance of all six array types.

**Results:**

The array combining all three types of elements (TP coils, DRs, and dipole antennas) provided the highest overall Rx performance; MR phantom experiments showed that integrating DRs with TP coils increased peripheral SNR by 250% and central SNR by 23% (for a total 38% gain in the center when also using dipole antennas in Rx). Human voxel model simulations confirmed that similar SNR gains can be achieved in vivo. Integrating DRs into TP coils also increased central Tx field efficiency by 4.6% and reduced the peak SAR_10g_ by 25.8% in the human voxel model Hugo.

**Conclusion:**

DRs and dipole antennas can significantly improve the overall Rx performance of TP coils. This concept can benefit MRI of the human lower extremity at 7 Tesla and encourage exploration of its utility for other clinical applications.

**Supplementary Information:**

The online version contains supplementary material available at 10.1007/s10334-026-01323-0.

## Introduction

Spatial resolution and acquisition time in MRI greatly depend on the radio frequency (RF) coils' receive (Rx) sensitivity [1, 2]. The existing RF technology relies on inflexible, multi-channel Rx arrays composed of conductive, resonant, loop-like elements and designed to accommodate a sufficiently large population of human subjects. Unfortunately, this seems to be a suboptimal solution for at least several clinical applications, such as musculoskeletal [3], pediatric [4], and breast MRI [5]. The size and geometry of those body parts can be highly heterogeneous, leading to reduced Rx performance due to suboptimal coil–sample distance. Increasing the filling factor of an Rx array by bringing its elements as close as possible to the region of interest for each patient could help significantly enhance SNR [6]. Therefore, high flexibility and inter-element decoupling are critical of the new generation of wearable Rx arrays. So far, several promising solutions have been proposed [3, 7–9]. The twisted-pair (TP) coil can be an excellent approach due to its robustness, self-decoupling, high level of flexibility, and the design’s simplicity and low cost [9–12]. Simultaneously, considerable progress has been made in developing other promising strategies to enhance Rx sensitivity, such as high-permittivity materials (HPMs) and dipole antennas, which could complement flexible RF coils and potentially deliver further SNR gains.

The role of HPMs in the design of modern MRI receivers is steadily growing, and recent advances in ceramic engineering encourage research in this direction [13]. Currently, HPMs can be manufactured in a wide range of electrical properties (*ε*_r_ up to several thousand) [14]. The primary consequence of using such high *ε*_r_ values is an improved RF field, which can enhance Rx sensitivity in MRI [15]. In earlier studies, HPMs were mainly used as either passive (dielectric pads [16, 17]) or—when appropriately designed [18]—active (dielectric resonator antenna arrays—DRAs [19–22]) elements. Previous work at 3T [23] also showed that placing a non-resonant HPM at the center of a conventional loop coil with planar dimensions similar to those of the HPM can yield significant Rx sensitivity gains.

In addition, there is growing evidence that dipole antennas can help to approach the ultimate intrinsic SNR, especially at high fields [24]. Dipole antennas produce an orthogonal electromagnetic field pattern to one of the loop coils, and those elements can be combined elegantly and with very low inductive coupling, significantly increasing SNR [25–27]. It was also demonstrated that dipole antennas could be combined with DRAs (a combination known as a dipolectric antenna [28]), bringing substantial SNR gains.

We hypothesize that combining three types of elements—flexible twisted-pair coils, HPMs (designed as resonant structures), and transceive (TxRx) dipole antennas—could substantially boost Rx sensitivity, especially at higher Larmor frequencies, such as ~ 300 MHz. To the best of our knowledge, such a combination has not been investigated so far. Therefore, this study aims to develop a flexible, multi-channel TP coil array with integrated DRs and dipole antennas, and to determine whether this approach can enhance SNR in MRI at 7T.

## Methods

Three different types of elements (TP, DR, and dipole) were combined in six different array configurations and studied to determine the optimal Rx performance in the context of a highly promising clinical application for wearable coils: lower extremity MRI at 7T. Since such combinations had not been compared previously, this strategy also aimed to gain further insights into how different sub-arrays interact. Hence, the following scenarios were investigated through both simulations and MR phantom experiments: four-channel TP coil array (4TxRx TP), four-channel TP coil array with four DRs (4TxRx TP + DR), four-channel dipole antenna array (4TxRx Dipole), four-channel dipole antenna array with four DRs (4TxRx Dipole + DR), eight-channel hybrid array comprising four TxRx dipole antennas, and four TxRx TP coils (8TxRx) and the same hybrid array with four integrated DRs (8TxRx + DR).

To test our hypothesis, a cylindrical phantom comparable in terms of dimensions (diameter = 110 mm) and electrical properties (dielectric constant *ɛ*_r_ = 78, electrical conductivity *σ* = 0.65 S/m) to that of an average human lower extremity was chosen.

To provide more compelling evidence that this strategy can impact the envisioned potential clinical application, additional simulations in human voxel Hugo (and Duke, as in Supplementary Materials) were conducted for the two types of arrays used in the previous phantom simulations and MR experiments: 8TxRx and 8TxRx + DR. The positioning of each TP coil and DR was adjusted to ensure a close fit with Hugo’s (and Duke’s) calf.

### Single-element experiments: TP coil and DR

**Fig. 1:**
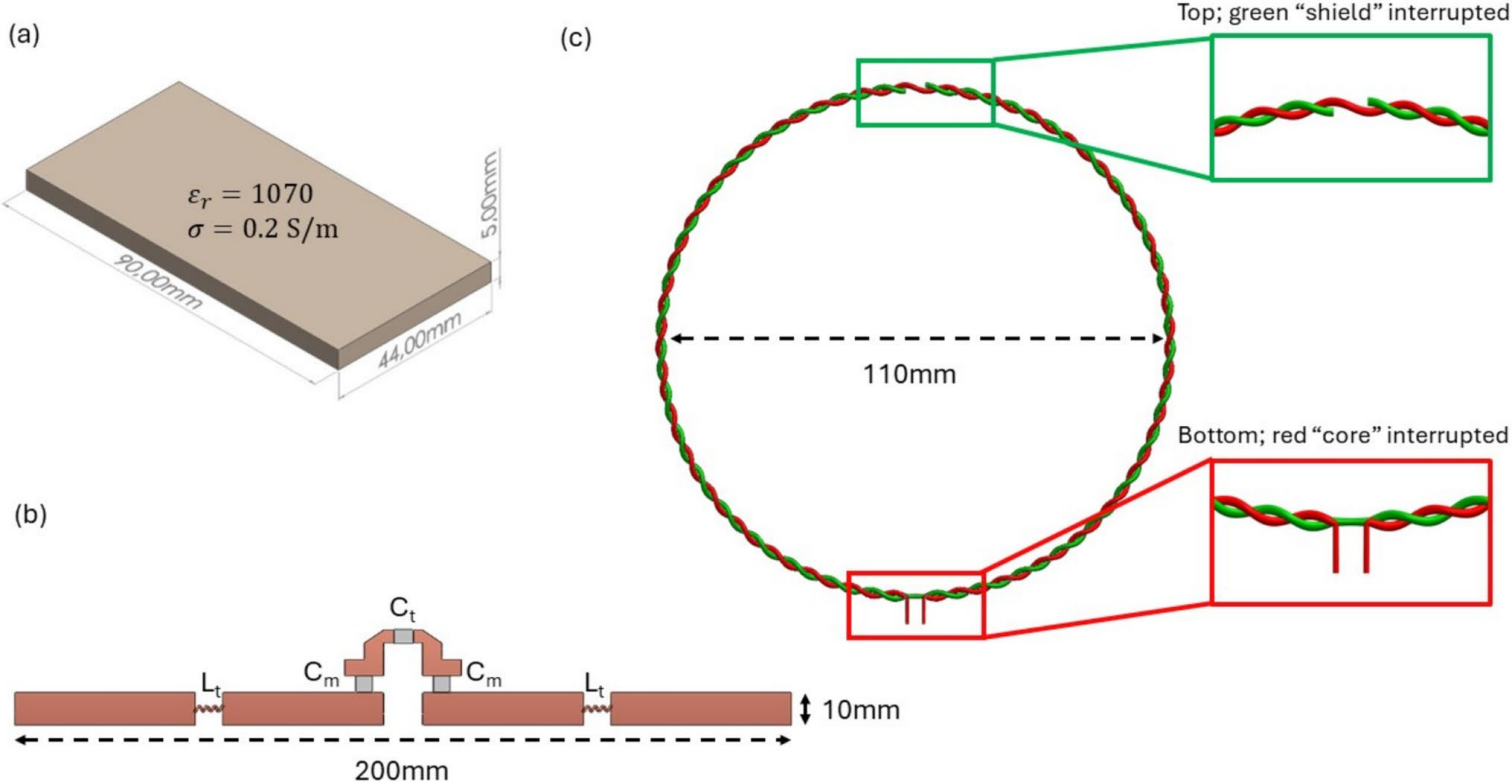
All three types of elements investigated in this study: **a** a dielectric resonator (DR; *ε*_r_ = 1070, σ = 0.2 S/m; thickness = 5 mm), **b** an inductively shortened dipole antenna (dipole); length = 200 mm; width = 10 mm; L_t_ = 210 nH; C_t_ = 16 pF; C_m_ = 1pF, and **c** a twisted-pair (TP) coil

**Fig. 2:**
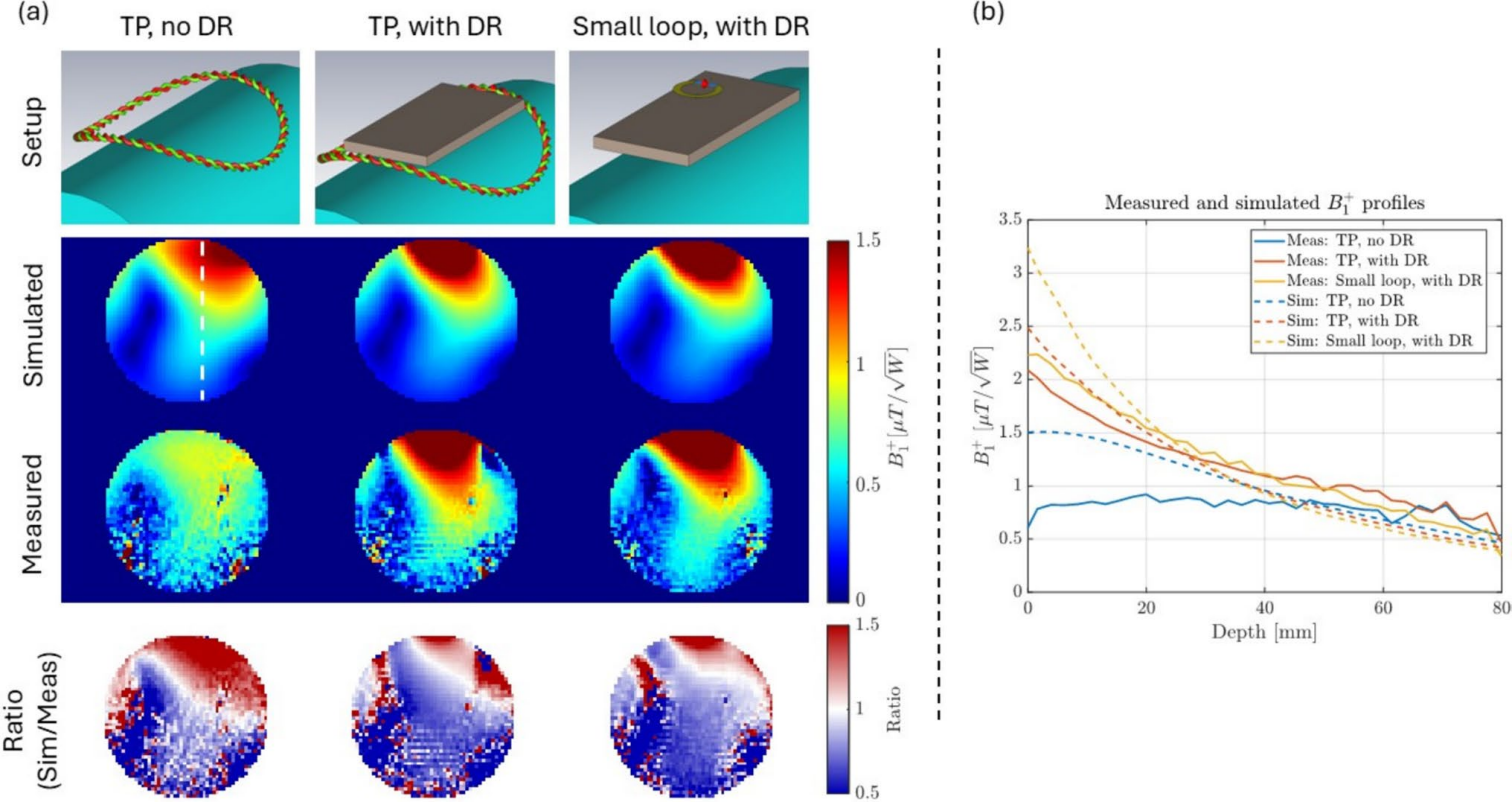
**a** Transmit field (B_1_^+^) distribution in a cylindrical phantom obtained for three single-element setups: TP coil, TP coil with an integrated DR, and a small loop-coupled DR, along with simulation/measurement division maps. The data obtained from numerical simulations were compared with MR phantom experiments at 7T. **b** Simulations and measurements showed that B_1_^+^ in the periphery (≤ 40 mm) was the highest for the small-loop coupled DR. Note that the DR is aligned in the same plane as the TP coil. At the same time, a small resonant loop element, inductively coupled with the DR, is separated from the DR by 6 mm

The TP coil was made by twisting two isolated (PTFE, *ɛ*_r_ = 2.1) wires (diameter = 1 mm) as described in the previous study [9] (Fig. 1). The cut at the top of each TP coil was approximately 5 mm. The diameter was 110 mm. A standard capacitive tuning and matching network was used to make the coil resonant at 297.2 MHz and power matched to 50 Ohms. A rectangular (90 mm × 44 mm × 5 mm) DR made of an HPM with a mass of 107 g, dielectric constant *ɛ*_r_ = 1070, and electrical conductivity *σ* = 0.2 S/m (HyQ Research Solutions, College Station, TX, USA) was used both in numerical simulations and MR phantom experiments. The resonance frequency of the low-order transverse electric (TE) mode for each DR was measured with a sniffer probe at ~ 315 MHz. The DR’s effect on the transmit field (B_1_^+^) efficiency was studied when placed at the center of the TP coil. In addition, a DR antenna using the same rectangular HPM block, with a small coupling loop (outer diameter = 18 mm) instead of the TP coil, was used as a reference design [28] as in Fig. 2.

### TP coil array with integrated DRs

**Fig. 3:**
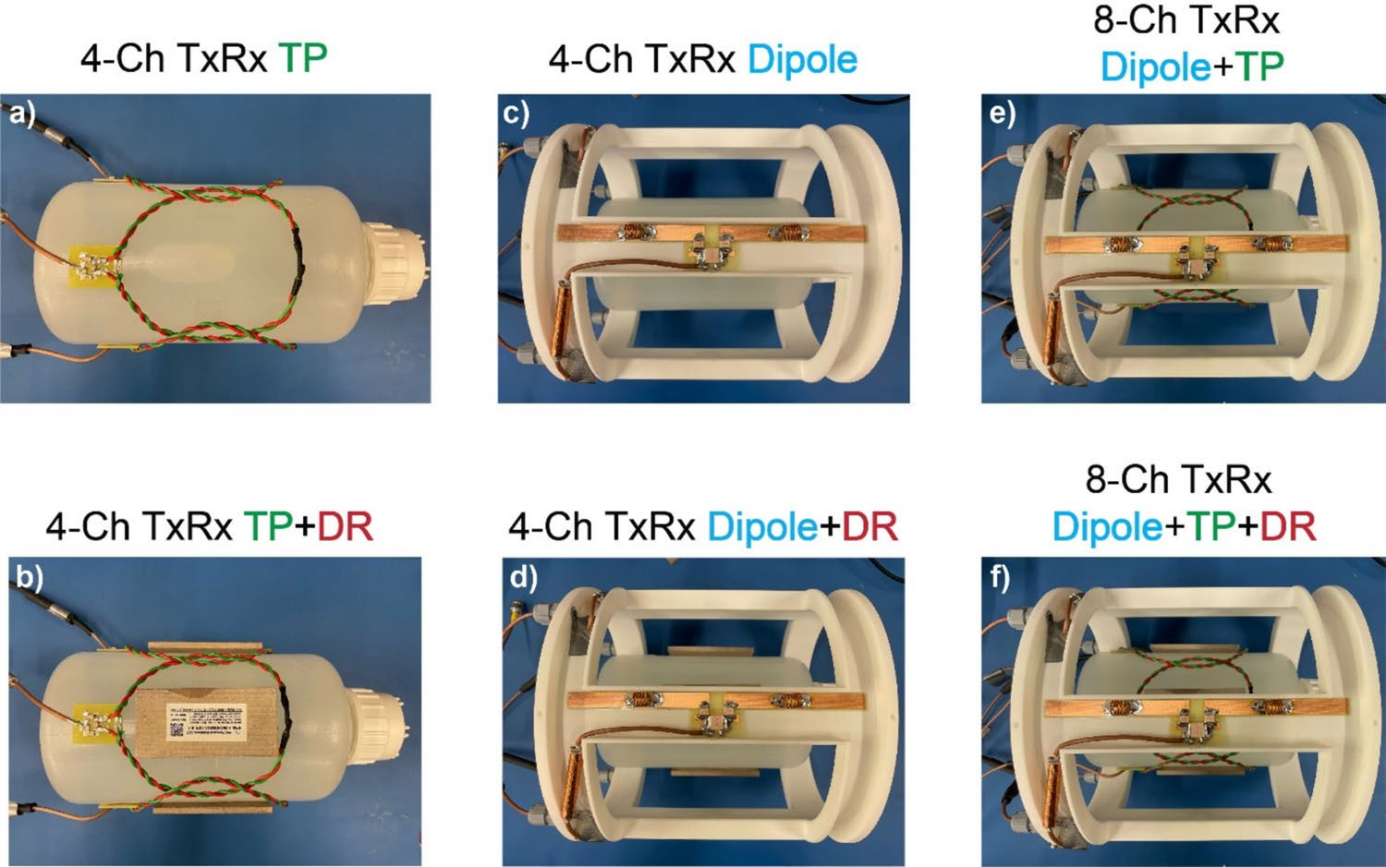
The photos of six multi-channel array configurations which were designed, constructed, and investigated in this study: **a** four-channel TxRx TP coil array without and b) with four DRs positioned in the center of each TP coil, c) four-channel TxRx dipole antenna array without and **d** with DRs positioned on the phantom, underneath each dipole antenna, **e** eight-channel TxRx dipole antenna and TP coil array, and **f** eight-channel TxRx dipole antenna and TP coil array with four integrated DRs. The distance between TP coils and dipole antennas was 38 mm

A four-channel TP coil array was constructed using four minimally overlapped (overlap/diameter ratio ~ 0.18) TP coils (Fig. 3). The array was tightly fitting to a cylindrical phantom (diameter = 110 mm, length = 200 mm) mimicking the human’s lower extremity. Each TP coil was tuned using a single capacitor in parallel (Ct: 1.0–2.1 pF) and two capacitors in series, ensuring balanced power matching (Cm: 3.6–8.2 pF). In the final array, four DRs were integrated, each positioned at the center of the TP coil. This required retuning each TP coil (Ct: 0.5–1.2 pF). During our experiments, double-sided tape was used to secure each TP coil and DR to the cylindrical phantom's plastic surface, ensuring the fixed position of each element.

### Dipole antenna array

A four-channel dipole antenna array suitable for lower extremity MRI at 7T was designed to complement the TP array. The dipole antennas were distributed concentrically (diameter = 200 mm) on a polyamide PA12, 3D-printed (EOSINT P 395, EOS GmbH, Germany; with selective laser sintering (SLS)) support. The dipole’s length was 200 mm and its width was 10 mm. The copper thickness was 35 µm. Each dipole was inductively shortened using two hand-wound solenoid coils (number of turns = 4, inner diameter = 8 mm, wire diameter = 1 mm; *L* = 156 nH (tuning range: 132–165 nH), which were placed in the center of each arm of the antenna. Each dipole was tuned and matched to 50 Ohm using a capacitive network (Ct = 10 pF, Cm = 56 pF) without variable capacitors, and connected to a T/R switch through a coaxial cable (K_02252_D, Huber + Suhner, Switzerland) with two bazooka cable traps (cylinder length = 50 mm; cylinder diameter = 9 mm) to improve rejection of common-mode currents (below – 35 dB) [28].

### Electromagnetic field simulations

Electromagnetic field simulations involving a cylindrical phantom and the Hugo human voxel model were performed in CST Microwave Studio 2025 (Dassault Systèmes, Vélizy-Villacoublay, France). In addition, to confirm our findings in Hugo, simulations in another human voxel model (Duke) using Sim4Life 8.0 (Zurich MedTech, Zurich, Switzerland) were performed and are included in the Supplementary Materials. The frequency domain solver with tetrahedral meshing was used in CST. The equation system solver accuracy of − 50 dB was used, along with an adaptive mesh enabled for a minimum of two and a maximum of five passes, and the default Gaussian excitation. The boundaries were set to open, spaced a quarter wavelength apart from the model. The number of tetrahedrons (mesh cells) was between 700.000 and 900.000 for all simulations. Simulations were performed on a Dual-Socket (2P) server equipped with two Intel Xeon E5-2600 v4 series processors (10 cores each) and 64 GB of DDR4 RAM. No GPU was used, as frequency-domain simulations are run exclusively on the CPU in CST. For the single-channel simulation, the time was about 30 min; for the four- and eight-channel arrays on the phantom, about 1–1.5 h. The human voxel model simulations took about 6 h. To evaluate transmit (B_1_^+^) and specific absorption rate (SAR), the results were normalized to 1 W stimulated power. SNR was calculated using the approach applied in the previous study [28]. Since our phantom simulations were directly compared to experiments, RF chain losses between the coil plug and the coil’s feed (T/R switches, power splitters, cables, cable traps) were measured using a network analyzer, exported as a Touchstone file, and directly incorporated into the CST co-simulation. Human voxel model simulations did not include RF chain losses. RF shimming for Hugo (and Duke in Supplementary Materials) was performed using a particle-swarm optimization method, developed earlier in our laboratory [29]. The algorithm did not consider local SAR minimization; only B_1_^+^ efficiency and homogeneity were considered in the cost function. Two different RF shims were compared: the axial RF Shim and the sagittal RF Shim. For each shim, a separate shimming region was defined (Fig. 7). The following B1 phase vectors were obtained (first four elements—dipole; other four—TPs): axial no DR = [148° 251° 0° 112° 0° 103° 202° 292°], axial with DR = [158° 256° 0° 110° 0° 97° 202° 274°], sagittal no DR = [125° 274° 306° 86° 0° 128° 171° 278°], sagittal with DR = [156° 294° 0° 108° 0° 137° 194° 309°].

### Bench evaluation and RF interface

Scattering parameter (S-parameter) matrices for all arrays were evaluated using a four-channel vector network analyzer (Keysight Technologies, USA). The arrays were loaded with a cylindrical phantom filled with water and NaCl (50 mM). The phantom’s dielectric constant (*ɛ*_r_ = 78) and electrical conductivity (*σ* = 0.65 S/m) were measured using a probe for dielectric properties measurements (DAKSY; Speag, Zurich, Switzerland). To interface the MRI scanner with every four-channel TxRx array investigated in this study, a two-stage 1:4 power divider (MRI.TOOLS, Berlin, Germany; phase offset <  ± 0.5°; peak power = 8 kW; average power = 100 W) was used along with four in-house developed T/R switches and four coaxial cables (two bazooka cable traps per cable) of appropriate lengths to produce a CP mode and connected to the array’s elements. The same T/R switch design was used in the eight-channel RF interface box used for the 8TxRx arrays [29].

### 7T MRI experiments

MR phantom experiments were performed on a whole-body 7T MR human scanner (Terra.X, Siemens Healthineers, Erlangen, Germany). For each array setup (Fig. 3), the transmit voltage amplitude in the center of the cylindrical phantom was adjusted to reach the flip angle (FA) of 90°, measured using a Turbo-FLASH B_1_^+^ mapping technique [30]: TR/TE = 5000/2.27 ms, FA = 8°, FOV = 352 × 256 mm^2^, resolution = (2.0 × 2.0 × 2.0) mm^3^. The TFL-DICOM images were processed assuming that each pixel’s signal intensity equals 0.1*FA. Then, the *V*_rms_ value at the RFPA output was used for normalization, which is now *V*_ref_ in the GUI for Terra.X systems. The *V*_rms_ value was then scaled down due to RF losses (1.9 dB and extra 1.0 dB when the combiner is connected, for single-Tx mode). To generate SNR maps, the approach proposed by Kellman and McVeigh [31] was used, that is, a standard 2D-GRE acquisition (TR/TE = 3500/5 ms, FA = 45°, FOV = 256 × 128 mm^2^, resolution = 2.0 mm × 2.0 mm × 2.0 mm) was followed by one with the transmit voltage amplitude set to 0. The raw SNR maps were corrected for FA variations using the previously acquired TFL data and normalized to the sine(FA).

## Results

To find out how DRs can affect B_1_^+^ efficiency, a TP coil was loaded with a cylindrical phantom, and the DR was positioned in the center of the TP coil (Fig. 2). Simulations showed that the small loop-coupled DR provided the highest B_1_^+^ efficiency in the periphery. In contrast, the TP coil performed minimally better at greater depth (in simulations 6–13% higher vs. the other two elements at 60 mm). B_1_^+^ mapping experiments confirmed that the highest B_1_^+^ efficiency in the periphery was obtained for the loop-coupled DR. Still, the highest B_1_^+^ efficiency below 40 mm was observed for the TP–DR combination. Measurements showed that the peripheral B_1_^+^ efficiency of the TP coil was substantially lower than predicted by simulations. The B_1_^+^ gain at the 10-mm depth for the TP–DR combination vs. TP coil was 35% and 107% for the simulations and measurements, respectively; at the 40-mm depth, it was 1% and 30%, respectively.

**Fig. 4:**
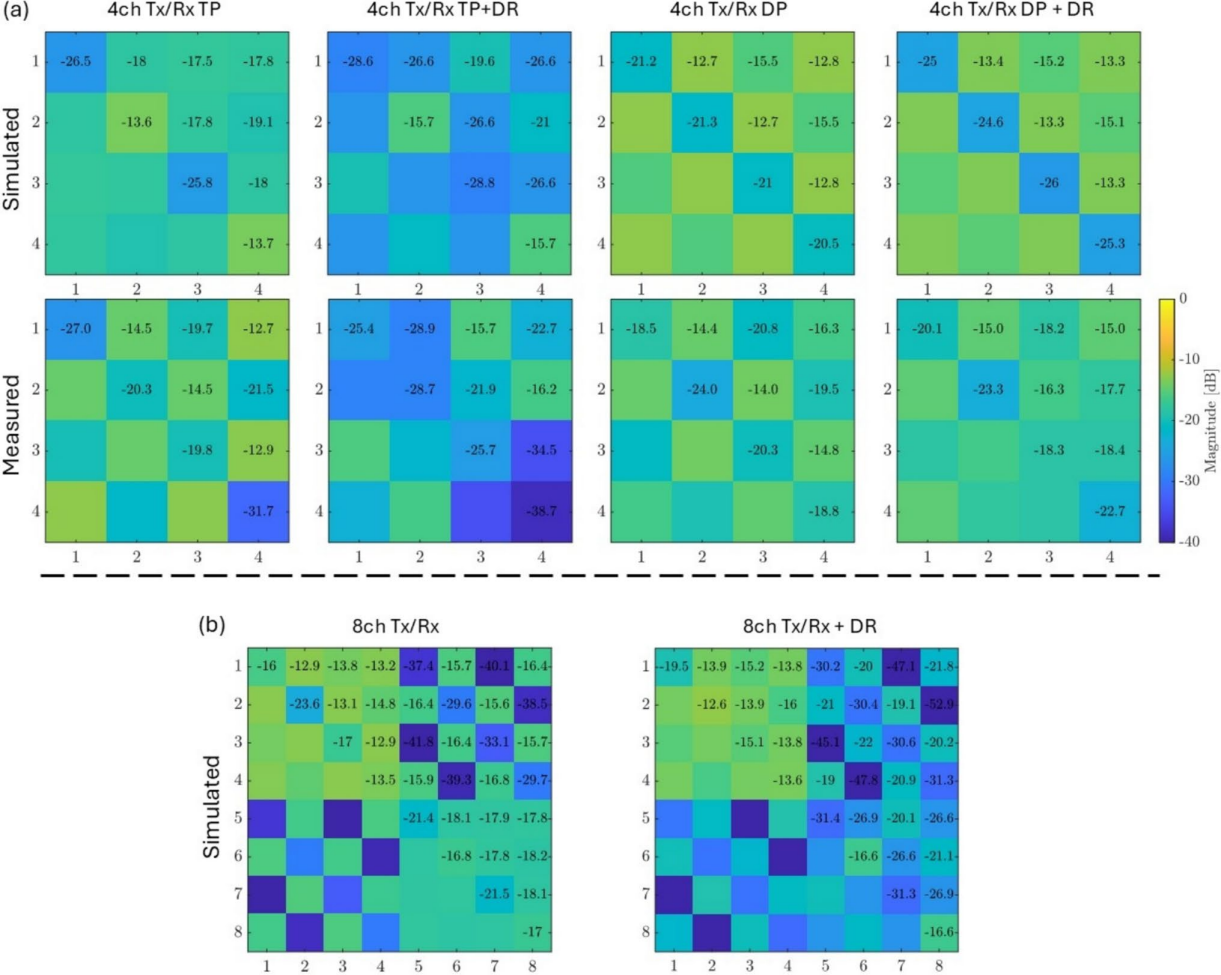
**A** Scattering parameter matrices for all six array configurations from Fig. 3 in both simulations and measurements. The TP coils and dipole antennas were lightly coupled without employing decoupling strategies. **B** For eight-channel configurations (elements 1–4—dipole antennas; elements 5–8—TP coils), coupling between different types of elements was very low without the need for critical overlapping (− 12.7 dB worst case for TP coils in measurements). The presence of DRs helped reduce coupling between TP coils to a remarkably low level (− 18.0 dB worst case in measurements)

The worst-case coupling between the elements of six different array configurations (Fig. 4) in simulations versus bench measurements was found to be: − 17.5 versus − 12.7 dB (4TxRx TP), − 19.6 versus − 15.7 dB (4TxRx TP + DR), − 12.7 versus − 14.0 dB (4TxRx Dipole), and − 13.3 versus − 15.0 dB (4TxRx Dipole + DR). For eight-channel configurations, the worst-case coupling was: − 12.9 versus − 13.1 dB (dipole–dipole—without DRs), − 13.8 versus − 16.0 dB (dipole–dipole—with DRs); − 17.8 versus − 12.7 dB (TP–TP—without DRs), − 20.1 versus − 18.0 dB (TP–TP—with DRs); − 15.6 versus − 19.7 (TP–dipole—without DRs), − 19.1 versus − 14.9 dB (TP–dipole—with DRs).

**Fig. 5:**
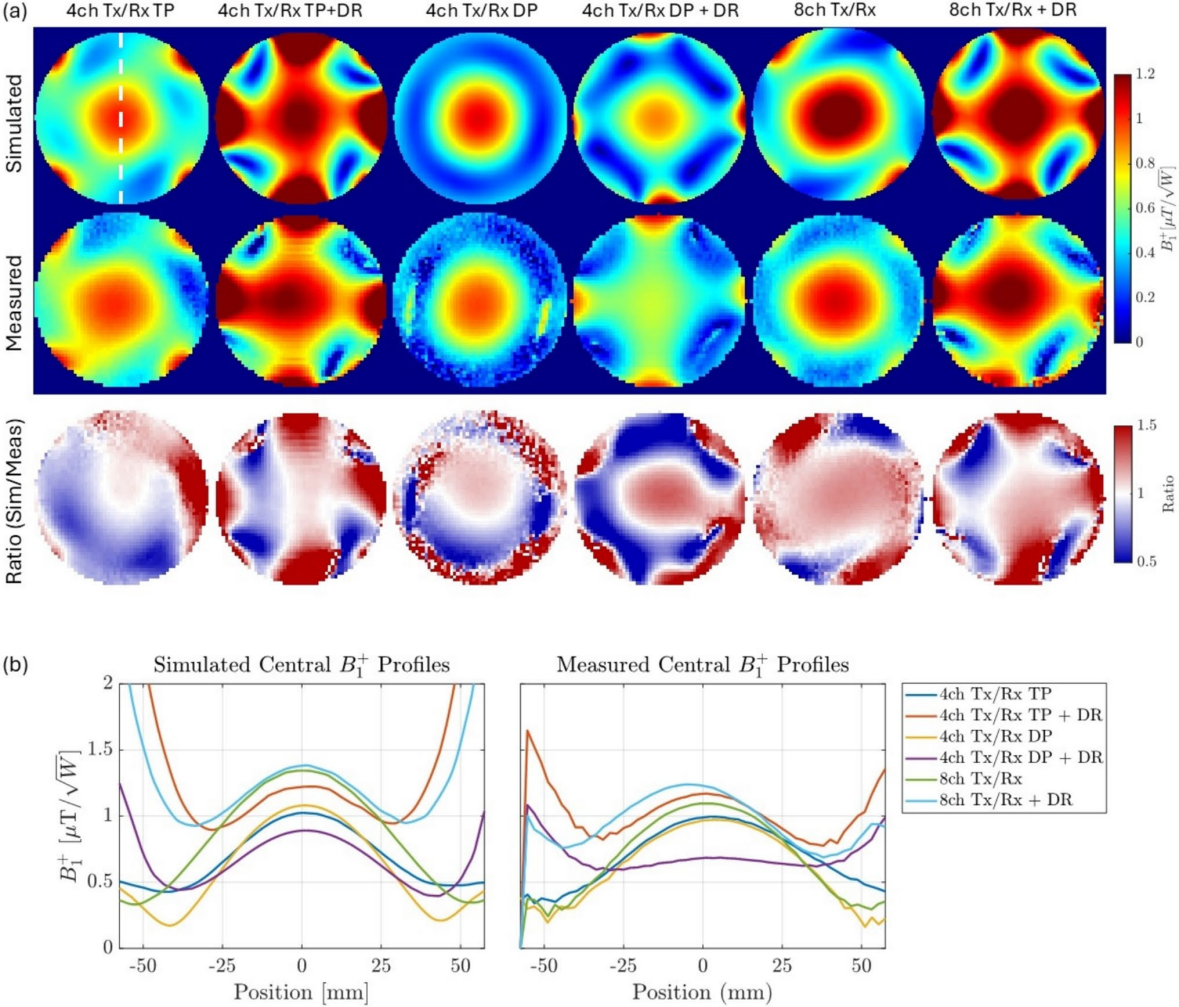
Simulated (RF chain losses included) and measured B_1_^+^ efficiency for all six arrays from Fig. 3 in the cylindrical phantom (central axial slice). The most apparent difference between simulated and measured central B_1_^+^ efficiency (22%) was found for the 4TxRx DP + DR. The most significant peripheral B_1_^+^ discrepancy was observed in two arrays that used TP coils coupled with DRs, likely due to TP coil losses primarily affecting peripheral B1+, as shown in Fig. 2

Numerical simulations showed that the combination of TP coils and DRs provided the highest central B_1_^+^ efficiency (1.22 µT/√W) when only four-channel arrays were used in Tx (Fig. 5). However, it was the 8TxRx with DRs, which provided the highest B_1_^+^ efficiency in the center among all designs (1.38 µT/√W). This finding was confirmed by MR experiments yielding 1.17 µT/√W in the center of the phantom for 4TxRx TP + DR, and 1.22 µT/√W for the 8TxRx with DRs.

**Fig. 6:**
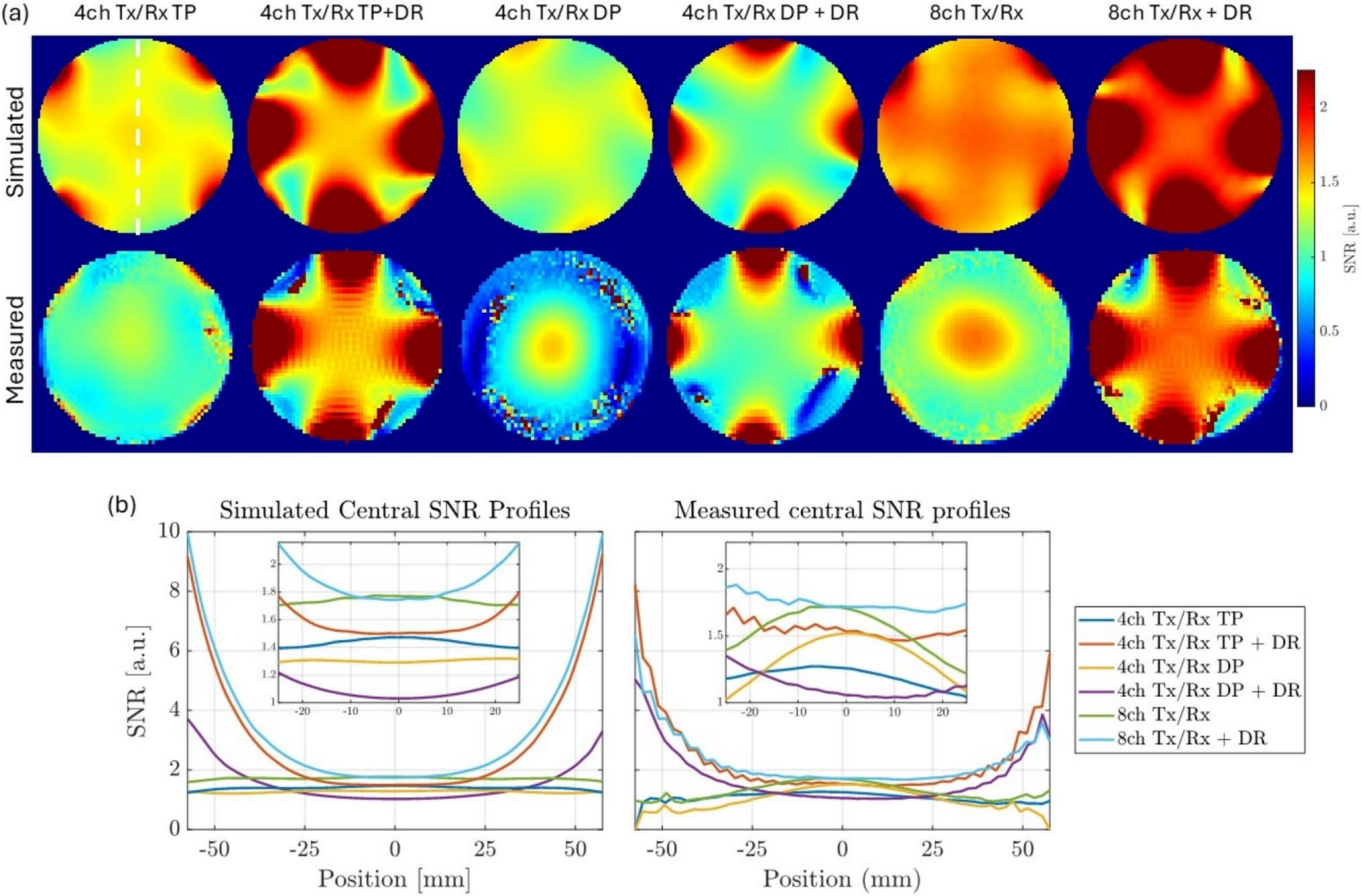
Simulated and measured SNR distribution for all six arrays from Fig. 3 in the cylindrical phantom (central axial slice). Measurements confirmed that using TxRx dipole antennas increased central SNR (see 8TxRx and 8TxRx + DR). At the same time, incorporating DRs resulted in substantial peripheral SNR gains with no central SNR loss when combined with TP coils in Rx. The most apparent qualitative difference was observed for 4Ch TxRx DP. The observed central SNR brightening was most likely due to a very low peripheral B_1_^+^ in contrast to the central B_1_^+^ (simulated, individual B_1_^−^ maps do not depend on the quality of B_1_^+^ as in MR experiments)

SNR calculations showed that the 8TxRx arrays with integrated DRs provided the highest overall SNR, outperforming the mean, in-axial-slice SNR of the other 8TxRx array by 62%. Experimental SNR results confirmed this trend; the 8TxRx array with DRs had the highest mean, in-axial-slice SNR, 62% higher than the no-DR version (see Fig. 6 and Tab.1).

**Table 1:** Summary of central B_1_^+^ efficiency (single value) and in-slice averaged SNR from simulations and measurements illustrated earlier in Figs. 5 and 6

	4ch Tx/Rx TP	4ch Tx/Rx TP + DR	4ch Tx/Rx DP	4ch Tx/Rx DP + DR	8ch Tx/Rx	8ch Tx/Rx + DR
Central $B_{1}^{ + } \;{\mathrm{value}}\;{[}\mu {\mathrm{T/}}\sqrt {\mathrm{W}} {]}$						
Simulated	1.02	1.22	1.08	0.89	1.34	1.38
Measured	0.99	1.17	0.97	0.68	1.09	1.22
Slice SNR mean [a.u.]						
Simulated	1.37 ± 0.12	2.04 ± 0.96	1.25 ± 0.10	1.28 ± 0.32	1.69 ± 0.10	2.74 ± 1.00
Measured	1.04 ± 0.14	1.69 ± 0.75	0.73 ± 0.33	1.16 ± 0.46	1.18 ± 0.23	1.91 ± 0.67

Measurements confirmed the central SNR trends observed in the simulation, except for the SNR of the 4TxRx TP in the center, which was reduced by approximately 25% compared with simulations (mean in-slice SNR: 21% difference), similar to Fig. 2.

**Fig. 7:**
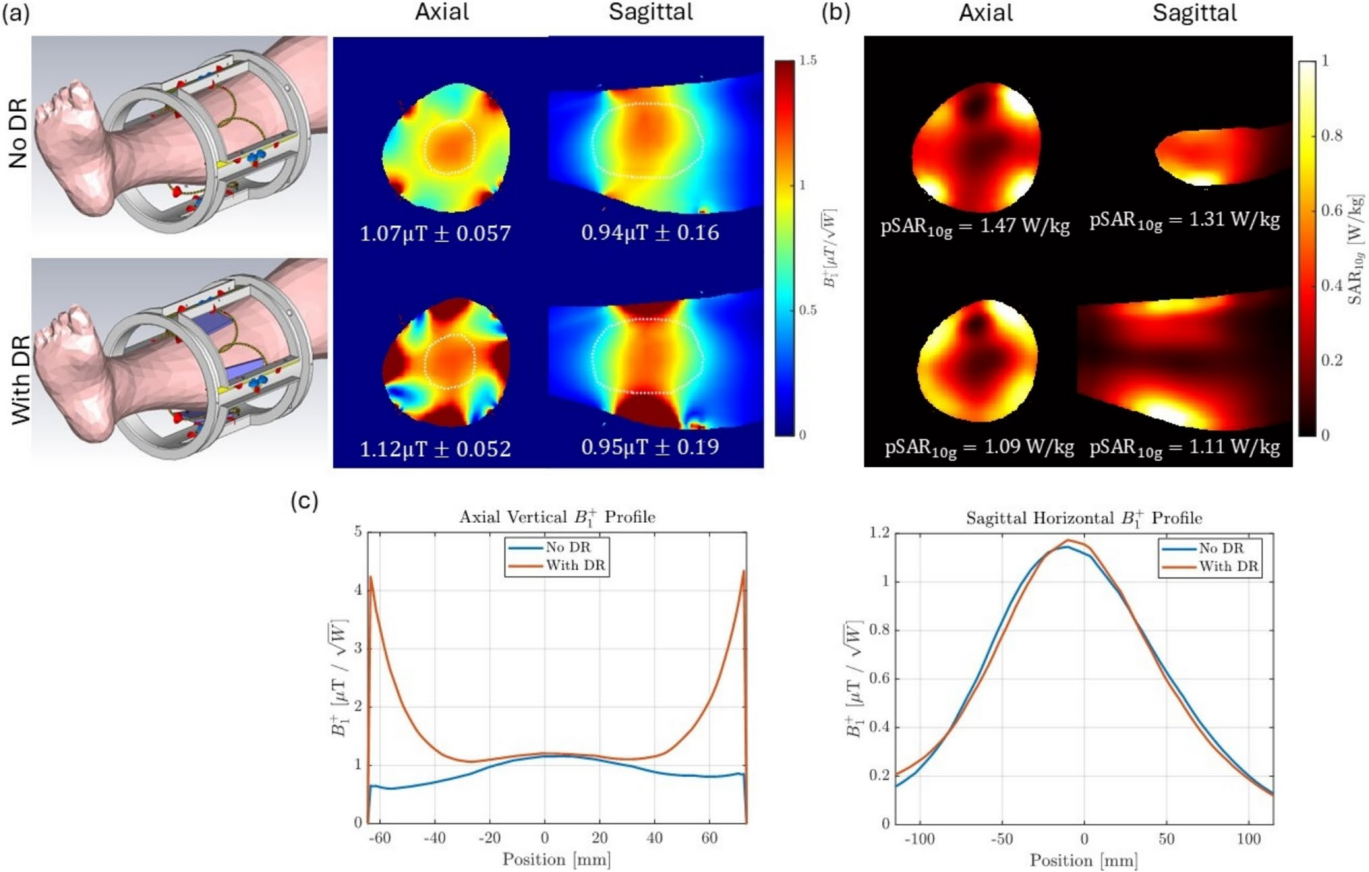
**a** Simulated B_1_^+^ efficiency (without RF chain losses) in the lower extremity (calf) of the human voxel model Hugo for two separate RF shims for each 8TxRx array: axial RF shim (shown only in the axial slice) and sagittal RF shim (shown only in the sagittal slice); with both shimmed ROIs highlighted using white dashed contours. The axial RF shim for the array with DRs increased B_1_^+^ efficiency in the ROI by 4.6% and B_1_^+^ homogeneity by 9.6%. B) Local SAR_10g_ distribution for both Axial and Sagittal RF shims, showing reduced pSAR_10g_ by 25.8% and 26.2%, respectively, when DRs were present. The slices shown in the Figure correspond to the ones with the highest pSAR_10g_

**Fig. 8:**
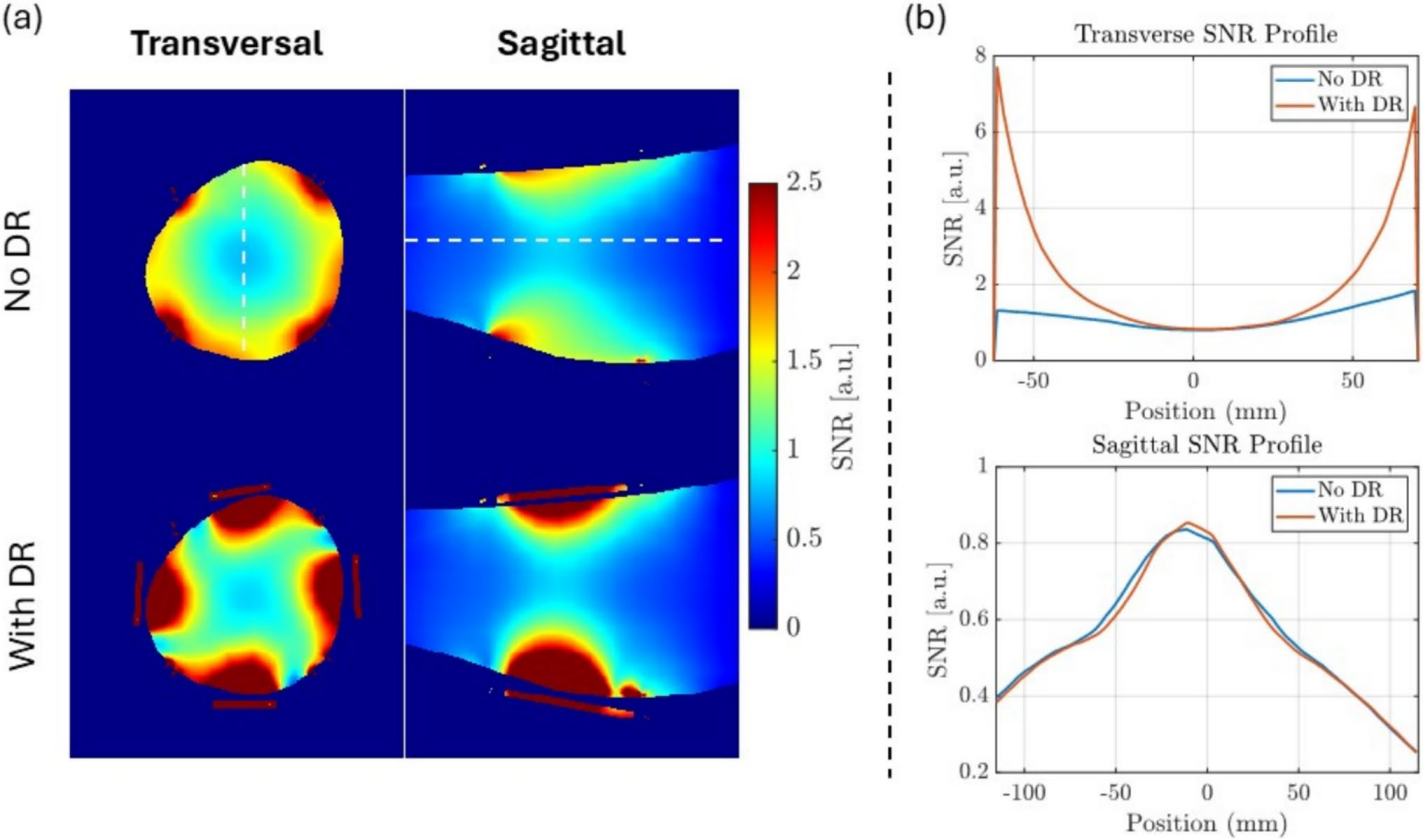
**a** Simulated SNR distribution in the lower extremity (calf) of the human voxel model Hugo, shown in the central axial and sagittal slices. **b** The data showed that using DRs provided a substantial increase in peripheral SNR (400%), similar to that in Fig. 6, with comparable SNR in the center

Electromagnetic field simulations in the lower extremity (calf) of the human voxel model Hugo showed that the central B_1_^+^ efficiency for the 8TxRx array with DRs was slightly higher (4.6%) versus the 8TxRx array without DRs for the “axial RF shim”, and comparable (1% difference) for the “sagittal RF shim” (Fig. 7). Both RF shims yielded lower pSAR_10g_ for the 8TxRx array with DRs: 12.2% (axial RF shim) and 18% (sagittal RF shim). Despite substantial SNR gains between the peripheral and the intermediate points (400% and 50%) in the axial slice, there was no significant change in the central SNR (Fig. 8). The average in-axial-slice SNR gain for the 8TxRx + DR was 45.4%.

## Discussion

This study demonstrates for the first time that combining flexible TP coils with resonant dielectric blocks and TxRx dipole antennas is feasible and can provide substantial Rx sensitivity gains for MRI at 7T. We investigated multiple combinations of TP coils, DRs, and dipole antennas and found that the 8TxRx array with DRs provided the best overall Rx performance, which was validated through both simulations and MR experiments (Fig. 6, 8).

The single-element data showed that mixing TP coils with DRs can provide a significant B_1_^+^ efficiency (equivalent to Rx sensitivity) increase versus their counterparts without any DR. This effect was not only present in the periphery, but also at greater depth than reported earlier at 3T for non-resonant HPMs [23]. The observed Rx sensitivity gains for a TP coil mixed with a DR can be explained by looking at the behavior of a loop-coupled DRA (Fig. 2). It becomes clear that the DR dominates the peripheral B_1_^+^ pattern when combined with the TP coil. At greater depth, the contribution from the TP coil is more apparent.

Although this study focused on Rx performance, it was an excellent opportunity to investigate Tx profiles of various designs. Phantom simulations and experiments showed that combining TP coils and DRs can provide impressive B_1_^+^ efficiency gains (4TxRx TP + DR vs. other 4TxRx arrays; see Fig. 5). Furthermore, the combination of all three types of elements: TPs, DRs, and dipoles (8TxRx) provided the highest central B_1_^+^ efficiency (Fig. 5) in our simulations involving a cylindrical phantom (13% higher than the TP–DR combination without dipoles). The idea of using dipole antennas as TxRx elements remains worth exploring, especially for applications that require substantial B_1_^+^ coverage in the z-direction. Indeed, we found that driving four dipole antennas in TxRx mode with integrated DRs can provide satisfactory z-coverage and slightly increase B_1_^+^ homogeneity within a sagittal region of interest in the cylindrical phantom (1.12 ± 0.11 uT versus 0.97 ± 0.08 uT for the dipoles without and with DRs, respectively—Supplementary Fig. 1).

While SNR in the center of Hugo’s calf was identical with and without DRs (Fig. 7), we observed SNR gain in peripheral (400%) and intermediate (50%) points for the array with DRs. Those results were compared with separate simulations in the human voxel model Duke using Sim4Life (see Supplementary Fig. 2); although we used different RF shim vectors for Duke and obtained different local SAR distributions, our findings in Hugo regarding B_1_^+^ efficiency and SNR were largely confirmed. Although our simulations showed that using DRs with TPs can indeed help reduce pSAR_10g_ (Fig. 7), one may be concerned about local SAR maintenance, especially when multiple DRs are positioned differently for different anatomies. To address potential RF safety concerns, we could use detuning strategies for DRs [32] during RF transmission. This solution, however, would also slightly detune dipole antennas; hence, it would be more convenient to use them in Tx-only, not TxRx. Additionally, one could consider performing thorough in silico modeling, assuming different positions of DRs versus different anatomies, and defining conservative safety margins for this approach.

The MR phantom measurements agreed reasonably well with the simulations and confirmed the predicted central SNR trends. The most apparent discrepancies likely resulted from inadequate loss modelling of TP coils and dipole antennas. TP coil modeling assumed perfect twisting of both wires, which has a critical impact on current distribution, but this was not fully achieved in the final design used in our experiments. One potential solution to this problem could involve identifying commercially available TP transmission lines for use in coil fabrication (for instance, an Ethernet cable). Although differences between measurements and simulations are not surprising, given previous reports on realistic losses in RF coils for UHF-MRI [33, 34], future work will undoubtedly contribute to a better understanding of loss mechanisms in custom-made TP coils at 7T.

Integrating DRs with TP coils helped to reduce the TP–TP coupling (Fig. 4) and stayed in agreement with previous findings, [35] demonstrating that TE modes excited in DRs alter the electromagnetic field distribution, which is more “focused” compared to loop coils. This effect was additionally boosted by a non-zero loss tangent (in our study *σ* = 0.2 S/m), leading to further coupling reductions. Note that it would not be possible to achieve this if TP coils were replaced with traditional loop coils (suboptimal overlapping leading to significant coupling and, in the worst case, resonance peak splitting).

Although we believe that a four4-channel TP coil array with four integrated DRs was sufficient for our proof-of-principle study, future work should focus on increasing the number of TP coils and DRs, as well as finding new optimal configurations for different clinical applications, notably lower extremity MRI. In addition, redesigning the Tx coil array into a splittable one, enabling free positioning of patients’ lower extremities, will be relevant for future clinical use. Given remarkably low coupling in the investigated TP–DR and TP–DR–dipole arrays (Fig. 4), it is likely that doubling (or even quadrupling when an extra row of elements will be considered) the number of TPs and DRs will bring further SNR gains and address the issue of having regions with B_1_^+^/B_1_^−^ voids that affect, for instance, B_1_^+^ homogeneity.

Future work should also investigate TP–DR array performance as a function of coil loading. To shed light on this matter, in our study, we examined the reflection coefficients of the TP–DR array as a function of distance from the cylindrical phantom (Supplementary Fig. 3). Although promising, the results indicate that developing a pair of arrays tailored to different loading conditions can be an optimal solution for lower extremity MRI at 7T. This, however, will have to be confirmed in a more realistic scenario using a truly wearable design (e.g., flexible band) adopted for in vivo applications.

One might consider the observed gains related to the TP coil diameter being substantially larger than the DR’s size. We still expect substantial gains even for loop elements that would be with similar planar dimensions as DRs [20, 23, 28], making our strategy highly promising for smaller TP coils and Rx arrays with more elements.

To conclude, our study demonstrates that combining wearable TP coil arrays with DRs and dipole antennas can yield significant SNR gains for MRI at 7T. This approach is auspicious for lower extremity MRI at 7T, but it can also be tailored for other clinical applications.

## Supplementary Information

Below is the link to the electronic supplementary material.

Supplementary file1 (DOCX 1944 kb)

## Data Availability

The data sets generated during the current study are available from the corresponding authors upon reasonable request.
