# Correction: Enhancing SNR in MRI at 7T using wearable coils, dielectric resonators, and dipole antennas

**DOI:** 10.1007/s10334-026-01332-z

**Published:** 2026-03-18

**Authors:** Daniel Wenz, Jules Vliem, Elizaveta Shegurova, Mark Widmaier, Lijing Xin, Dimitrios C. Karampinos, Irena Zivkovic

**Affiliations:** 1 CIBM Center for Biomedical Imaging, Lausanne, Switzerland; 2 MR Imaging and Technology, EPFL Swiss Federal Institute of Technology, Lausanne, Switzerland; 3 Department of Electrical Engineering, Eindhoven University of Technology, Eindhoven, The Netherlands; 4 Laboratory of Magnetic Resonance Imaging Systems and Methods, EPFL Swiss Federal Institute of Technology, Lausanne, Switzerland; 5 Institute of Physics (IPHYS), EPFL Swiss Federal Institute of Technology, Lausanne, Switzerland

**Correction: Magnetic Resonance Materials in Physics, Biology and Medicine** 10.1007/s10334-026-01323-0

In the original version of this article, the acknowledgements section was incomplete and should have read “The authors acknowledge access to the facilities and expertise of the CIBM Center for Biomedical Imaging, a Swiss research center of excellence founded and supported by Lausanne University Hospital (CHUV), University of Lausanne (UNIL), Ecole polytechnique federale de Lausanne (EPFL), University of Geneva (UNIGE), and Geneva University Hospitals (HUG). We also acknowledge the MRI Platform of the FCBG (Fondation Campus Biotech Geneva). The authors would like to thank Jérémie Clément (Siemens Healthineers) and André Kühne (Magforce NT GmbH) for very helpful discussions during the manuscript’s revision process’’.

