# Supplementary material for: Enhancing SNR in MRI at 7T using wearable coils, dielectric resonators, and dipole antennas
Source: MAGMA. 2026 Jan 20;39(4):571–82. doi: 10.1007/s10334-026-01323-0 (PMC13447474; doi:10.1007/s10334-026-01323-0)
Supplement: Supplementary file 1 [file 10334_2026_1323_MOESM1_ESM.docx]

**Supplementary Figures**

**Supplementary Figure 1:** a) Simulated sagittal B_1_^+^ (CP mode) and SNR profiles for the cylindrical phantom from the data presented in Figures 5 and 6. Note that these simulations do not include the RF chain losses. The DR’s in-axial-slice focusing is apparent for 4ch TxRx TP+DR and 8ch TxRx + DR. Dipole antennas, as expected, provide excellent z-coverage; interestingly, coupling DRs with dipole antennas provided excellent longitudinal (z-axis) B_1_^+^ homogeneity, as in b). DRs clearly provide outstanding peripheral SNR gains. Adding dipole antennas in Rx increased in-axial-slice SNR, as did B_1_^+^, and sagittal, off-center regions were similarly enhanced.

**Supplementary Figure 2:** B₁⁺ simulations with corresponding 10 g-averaged SAR values and SNR maps in the lower leg (calf) of the human voxel model Duke. Simulations were performed in Sim4Life (ZMT, Zurich, Switzerland) using four twisted-pair (TP) coils and four dipole antennas operating at 297.2 MHz, with and without dielectric resonators (DRs). The finite-difference time-domain (FDTD) multiport solver was used. For the no-DR configuration, simulations comprised 149.6 million cells, each channel requiring approximately 7 h on an NVIDIA GeForce RTX 3090 GPU. For the with-DR configuration, the grid contained 154.6 million cells, each channel taking about 5 h on three NVIDIA H100 GPUs (~2 GB per device). A capacitive tuning and matching network with variable capacitors for TP coils was used to achieve resonance and 50 Ω matching. Transmit phases were optimized via an RF shimming algorithm, normalized to 1 W input power, and exported for visualization. The following phases were used for both shims (#Elements 1-4: TP coils; #Elements 5-8: Dipole antennas): No DR axial shim = [247° 0° 83° 133° 70° 185° 259° 50°]; DR axial shim = [0° 115° 190° 270° 187° 305° 352° 103°] as well as No DR sagittal shim = [0° 124° 181° 156° 135° 281° 311° 89°]; DR sagittal shim = [0° 151° 210° 356° 122° 295° 334° 96°].

**Supplementary Figure 3.** Bench measurements: loading sensitivity evaluation for the 4-ch TP/DR array. Reflection parameters S_11_, S_22_, S_33_, and S_44_ (in dB) were measured as a function of the distance between the 4-ch TP/DR array and the cylindrical phantom. The distance was increased by adding additional bubble wrap layers (4 mm thickness of a single layer). Before adding a new layer, TP coils and DRs were unstuck from the surface and restuck once the new bubble layer was provided. Note that this operation resulted in minimal overlap (4 mm) and progressed to a complete separation of ~5 mm between the TP coils (24 mm). Initial tuning and matching (below -15 dB) were performed at a distance of 4 mm. Measurements using a 4-channel network analyzer were performed at 5 other distances up to 24 mm. Increasing the distance from 4 to 24 mm, resulted in increased reflected power at 297.2 MHz from 1.7% to 31.6% (S_11_), from 2.8% to 34.7% (S_22_), from 1.4% to 46.7% (S_33_), and from 1.8% to 25.0% (S_44_). Coupling between TP coils only changed for the 8-mm distance due to the overlapping (directly on top of each other) parts of the TP coils.

**Supplementary Figure 4:** a) Zoomed-in tuning/matching circuit for the TP coil used in 8TxRx experiments. b) RF interface used for single-element experiments in Fig. 2; only one T/R switch (green color) was used. c) 8-channel RF interface box (8 custom-built T/R switches) used in 8TxRx experiments. d) 4-channel RF interface (two-stage 1:4 power divider, 4 T/R switches, CP mode phase cables) used in 4TxRx experiments.
